# Doubts About the Role of Rehearsal in the Irrelevant Sound Effect

**DOI:** 10.1027/1618-3169/a000527

**Published:** 2021-12-15

**Authors:** Jamielyn R. Samper, Alexandra Morrison, Jason Chein

**Affiliations:** ^1^ Department of Psychology, Temple University, Philadelphia, PA, USA; ^2^ California State University, Sacramento, CA, USA

**Keywords:** irrelevant sound effect, rehearsal, strategies, working memory

## Abstract

**Abstract.** The irrelevant sound effect (ISE) describes the disruption of processes involved in maintaining information in working memory (WM) when irrelevant noise is present in the environment. While some posit that the ISE arises due to split obligation of attention to the irrelevant sound and the to-be-remembered information, others have argued that background noise corrupts the order of information within WM. Support for the latter position comes from research showing that the ISE appears to be most robust in tasks that emphasize ordered maintenance by a serial rehearsal strategy, and diminished when rehearsal is discouraged or precluded by task characteristics. This prior work confounds the demand for seriation with rehearsal. Thus, the present study aims to disentangle ordered maintenance from a rehearsal strategy by using a running memory span task that requires ordered output but obviates the utility of rehearsal. Across four experiments, we find a significant ISE that persists under conditions that should discourage the use of rehearsal and among individuals who self-report use of alternative strategies. These findings indicate that rehearsal is not necessary to produce an ISE in a serial recall task and thus fail to corroborate accounts of the ISE that emphasize the involvement of rehearsal.


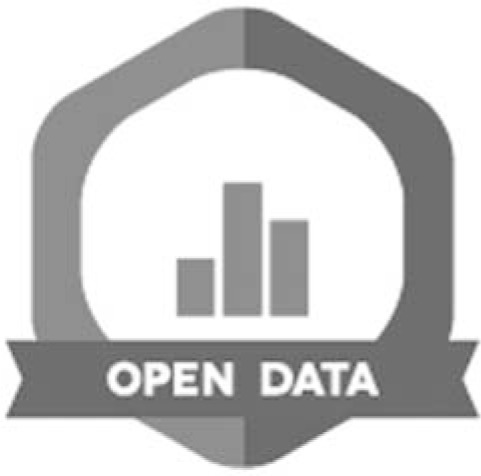


The irrelevant sound effect (ISE) occurs when extraneous background sounds disrupt memory and information processing, most typically measured in the context of serial short-term memory task performance (e.g., Colle & Welsh, 1976; Hughes et al., 2005; Jones et al., 1992; Lange, 2005; Salamé & Baddeley, 1982). Investigations of this phenomenon inform our understanding of why and how cognition may be diminished in noisy environments (Banbury & Berry, 1997; Beaman, 2005) and also provide a testing ground for the evaluation of alternative theories of working memory (WM; Chein & Fiez, 2010; Neath, 2000; Oberauer et al., 2018), the temporary mental workspace thought to underpin a very wide range of higher cognitive abilities. Indeed, there has been considerable debate surrounding the mechanisms through which the ISE arises (Bell et al., 2019a, 2019b; Hughes, 2014; Jones et al., 1992; Jones & Tremblay, 2000; Lange, 2005; Le Compte, 1994; Marsh et al., 2009), reflecting deep-seated theoretical differences in the conceptualization of WM and its central machinery.

Recent work has focused on arbitrating between alternative process-based explanations for the ISE, which attribute the ISE to disruption (caused by background sounds) of specific mechanisms thought to be instrumental for encoding and maintaining information in WM (Jones, 1994; Jones et al., 1992; Jones & Tremblay, 2000; Marsh et al., 2008).

Process-based interference explanations can generally be classified into two camps: accounts attributing the ISE to attentional mechanisms and accounts attributing the phenomenon to disruption of the processes that support ordered maintenance of memoranda, namely serial rehearsal. Attentional accounts contend that disruption occurs due to the split allocation of attentional resources to to-be-remembered (TBR) and to-be-ignored (TBI) objects. The transient redirection of attention away from TBR items may happen as a momentary attentional capture (Cowan, 1995; 1999) or in a graded fashion (as in the *graded attentional model*; Bell et al., 2019; Röer et al., 2014a; Schröger et al., 2000). Attentional explanations for the ISE garner support from studies showing habituation to irrelevant sounds (ISs; Banbury & Berry, 1997; Bell et al., 2012; Kattner & Ellermeier, 2020; Röer et al., 2011, 2014a), work linking forms of the ISE to an attentional orienting response (Nöstl et al., 2012; Parmentier et al., 2011; Röer et al., 2014a; Vachon et al., 2012), and investigations of the neural correlates of the ISE (Chein & Fiez, 2010; Röer et al., 2014a).

An alternative, and influential, class of models has been broadly referred to as *order-interference* accounts (Beaman & Jones, 1998; Jones et al., 1996; Jones & Macken, 1993; Jones et al., 1992). These accounts assume that the ISE arises due to conflict between the preattentive and obligatory processing of a sound stream for order and the seriation processes used to encode and maintain the TBR stimuli in their correct presentation order (Beaman & Jones, 1997; Campbell, 2000; Jones, 1992, 1993; Jones et al., 2010; Jones & Tremblay, 2000; Macken et al., 1999). The *changing-state hypothesis* further refines this account by asserting that the acoustic changes between successive objects in a sound stream are what cause it to be processed as an ordered sequence, thus explaining why more highly varied (changing-state) sound streams are significantly more disruptive of ordered maintenance than sound streams constructed of repeated auditory objects (a steady-state stream) – a phenomenon known as the changing-state effect (Beaman & Jones, 1997; Jones, 1993; Jones et al., 1992).

Most models falling into this class, such as the Object-Oriented Episodic Record model (Jones, 1993; Jones et al., 1996), the Perceptual-Gestural account (Jones et al., 2004, 2006, 2007; Macken & Jones, 2003), and the Duplex-Mechanism account (Hughes, 2014; Hughes et al., 2013; Hughes & Jones, 2005; Hughes et al., 2005, 2007), further emphasize the strategic deployment of rehearsal as the primary seriation process associated with item maintenance. Under these models, the errors observed in serial short-term memory tasks are assumed to result from conflicting order cues produced by the processing of the IS stream and the covert serial rehearsal of TBR items (Beaman & Jones, 1997, 1998; Jones, 1993; Jones et al., 1996; Macken et al., 1999). We will hereafter refer to accounts making this specific additional assumption as *rehearsal-disruption* accounts, to contrast them with other order-interference models, such as the Token-Gradient model (Campbell, 2000; Campbell et al., 2003), that place less emphasis on the coupling between rehearsal and seriation.

Two sources of evidence are generally cited in favor of rehearsal-disruption accounts. First, ISs are known to disrupt recall performance even when the sounds are presented only during a postpresentation retention interval (Macken et al., 1999; Norris et al., 2004; Chein & Fiez, 2010, Experiment 2), indicating that the phenomenon can be isolated to mechanisms that are engaged specifically during retention. The second line of argument used to establish a link between serial rehearsal and the ISE forms the basis for the present investigation and derives from studies showing that the ISE is relatively diminished (or absent) in tasks that preclude or discourage serial rehearsal. For example, Beaman and Jones (1997) demonstrated a strong changing-state ISE when subjects were tested using a serial recall task, but a substantially diminished ISE when testing was conducted using recognition, paired associates, and missing-item tasks, for which serial rehearsal is presumably an ineffective, and less likely to be adopted, strategy. Hughes et al. (2007) replicated the absence of a changing-state effect in the missing-item task. Similarly, Henson et al. (2003) reported a large ISE (changing-state sounds compared to quiet control) in a list probe task expected to encourage serial rehearsal, but a weak (though significant) ISE in association with an item probe task that did not require order to be maintained and, hence, may have discouraged rehearsal.

While there is some consistency to the above findings on the task and strategy-dependent nature of the ISE, there are also some contradictory findings, and a growing number of studies finding significant ISEs in tasks for which rehearsal seems quite unlikely. A notable early exception comes from the work of LeCompte, who reported significant ISEs in a free recall (LeCompte, 1994), recognition (LeCompte, 1994), and missing-item task (LeCompte, 1996). In advocating the rehearsal-disruption account, Beaman and Jones (1997, 1998) countered that the findings of LeCompte might be explained by participants’ continued strategic use of rehearsal despite the absence of any explicit order requirements, as the effect can be eliminated when participants are required to perform articulatory suppression, an act of overtly repeating a series of words, letters, or syllables to prevent rehearsal. There are, however, several additional studies in which a significant ISE was found in a task for which engagement of rehearsal processes seems particularly unlikely. ISEs have been found, for instance, in tasks with no obvious short-term mnemonic component, including a sequence learning task (Gisselgård et al., 2007) and a continuous statistical learning task (Neath et al., 2009). A further study that bears mentioning in this regard is that of Stokes and Arnell (2012). In their study, the use of rehearsal was obviated in two ways. First, participants in the study completed a lexical decision task for a sequence of 200 items, which should be much too long to rehearse in any meaningful way. Second, participants were entirely unaware that there would be a later recognition memory test for the items and therefore had no incentive to engage in any intentional or effortful maintenance strategies (such as rehearsal). Nevertheless, changing-state and steady-state sound streams were found to significantly reduce the number of words accurately recognized on the surprise test compared to quiet control conditions, suggesting a general disruptive effect of sounds. This finding stands out in the literature as the only example where a significant ISE was reported in association with a task that neither emphasizes short-term/WM processes nor has an obvious seriation requirement.

## Dissociating Seriation and Rehearsal With the Running Memory Span Task

The apparently contradictory findings in relation to a rehearsal-disruption account call for further scrutiny of the ISE phenomenon and the specific conditions in which it arises. While failures to affirm the attentional account are sometimes proffered as support for the alternative rehearsal-disruption view (Beaman et al., 2014; Beaman & Jones, 2016), such support is plainly indirect. Meanwhile, work aiming to more directly test the predictions of the rehearsal-disruption perspective (e.g., Bell et al., 2012; Stokes & Arnell, 2012) is, to date, still quite limited.

Accordingly, the present study sought to expand on this small body of prior work by exploring the potential for an ISE to arise in a short-term memory task, the running memory span (RMS) task, that requires serial information processing but should (at least under certain conditions) strongly discourage serial rehearsal. Thus, using this task, we hoped to dichotomize the involvement of two potentially distinct componential processes that underlie serial rehearsal – *seriation* and *rehearsal* – noting that the link between each and the ISE may not be the same.

In a typical RMS task, a list of sequential stimuli is presented for an unpredicted duration, after which some number of items from the end of the list must be recalled (Pollack et al., 1959). The unpredictability of list lengths reduces the ability of participants to group and rehearse items (Hockey & Hamilton, 1977), and the typically fast speed of item presentation restricts the types of strategies that a participant can successfully deploy (Bunting et al., 2006; Hockey, 1973). In the work of Hockey (1973), a slower presentation rate led to better performance when participants were instructed to rehearse rather than passively listen, whereas with a faster presentation rate, participants performed better when asked to passively listen rather than rehearse. In a demonstration by Bunting et al. (2006), response times and subject self-reports confirmed that participants varied strategy choice depending on presentation rate. Thus, while slower presentation rates in an RMS task might lead participants to adopt a rehearsal strategy, faster presentation rates appear to promote a passive, more purely attention-based, strategy.

Accordingly, this task presents a useful platform to test whether rehearsal is a necessary component of the ISE. By taking advantage of the strategy differences that coincide with presentation speed, we hoped to observe whether task conditions under which participants are more and less likely to rehearse lead to differences in the magnitude of the ISE. Importantly, the RMS task maintains a seriation requirement, thus allowing us to manipulate rehearsal within a short-term memory task that still involves ordered memory.

In Experiment 1, the magnitude of the ISE is compared across two versions of the RMS task: a slower presentation format that makes rehearsal more likely and a fast presentation format in which rehearsal is a less likely strategy. Experiment 2 addresses a possible confound in Experiment 1 by eliminating the potential for prior exposure to a slow format RMS task to elicit a rehearsal strategy that may carry over to subsequent performance in a fast format condition. Experiment 3 investigated the role of rehearsal in the changing-state effect by comparing the ISE in conditions of changing-state sounds versus steady-state sounds (relative to silence). Finally, to illicit a potentially broader range of strategies, Experiment 4 used word stimuli instead of letters as the TBR items and assessed participants’ subjectively perceived use of strategies via an expanded strategy questionnaire (Morrison et al., 2016).

## Experiment 1

The aim of Experiment 1 was to isolate serial order task demands from rehearsal strategies using slow- and fast-paced versions of an RMS task and to thereby determine if an ISE emerges in conditions that make rehearsal more likely (slow version) and less likely (fast version). By comparing performance in quiet and IS conditions across these two task variations, we sought to determine if using rehearsal changes an individual’s susceptibility to the ISE.

### Method

#### Participants

Twenty-five native English-speaking, Temple University undergraduates (*M*_*age*_ = 19.25, 18 females) who reported normal or corrected-to-normal vision and hearing, and unimpaired use of their dominant hand participated in exchange for course credit. All subjects gave written informed consent. Data from only 23 participants were included in the final analyses because two participants failed to complete the full paradigm.

#### Stimuli

Visual stimuli were presented by using a computer at a viewing distance of about 50 cm. A list of 12, 14, 16, 18, or 20 letters was presented for each trial. The lists were constructed by random sampling from the English letters B, F, H, J, L, M, Q, S, and Y, with sampling restricted from repetition among the last six letters in the presented set. The letters were displayed sequentially, in uppercase, 18 point, white, bold, Arial font on a navy-blue background.

IS sequences were constructed using Audacity 1.2.6 (Verilogix Inc., Palos Verdes Peninsula, CA, USA) digital audio editing software and comprised the spoken digits 1 through 4, recorded in a male voice at 16-bit resolution and a sampling rate of 44 kHz, each edited to be exactly 350 ms long. Multiple sound sequences were constructed by sampling these recordings in a pseudorandom order, with the same digit never presented twice in succession. In each TBI sequence, a spoken digit occurred once every 500 ms (350 ms stimulus, 150 ms ISI). The irrelevant background sound sequence always began 500 ms before the first item in the TBR list was presented and terminated 500 ms after the TBR list ended, regardless of list length or presentation rate.

#### Design and Procedure

Participants were tested in a single session lasting under 1 hour. Each participant was assessed for performance in a variant of the RMS task adapted from Bunting et al. (2006). The task was created and administered using E-Prime 2.0 software (Schneider et al., 2002). For each participant, RMS accuracy was assessed in two separate blocks: one using the slow rate of TBR item presentation (1 item per second) and the other using the fast rate of TBR item presentation (3 items per second). The order of the fast-paced and slow-paced blocks was counterbalanced across participants. Each block consisted of four practice trials, followed by 30 experimental trials, half of which were tested under quiet conditions and the other half under concurrent presentation of an IS sequence that participants were instructed to ignore. Quiet and IS trials were randomly intermixed within each block.

**Figure 1:**
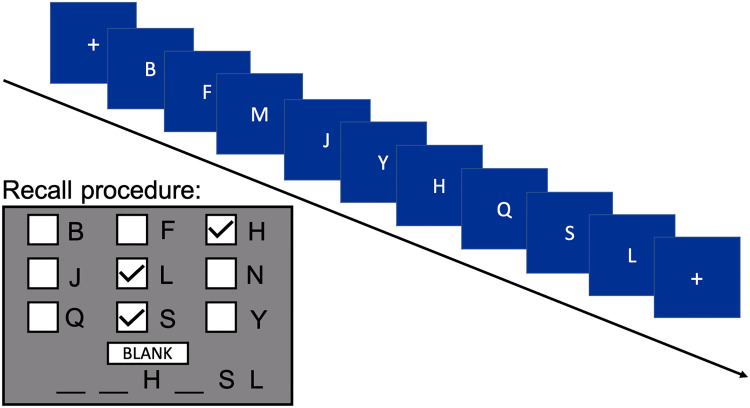
Running memory span task. Varied length sequences of 12–20 letters were presented at a speed of either one letter per second (slow condition) or three letters per second (fast condition). At the retrieval prompt, participants attempted to select the last six shown letters in their correct sequential order.

Each trial (Figure 1) was initiated by a mouse click, which was followed by a brief fixation marker (+, 1 seconds) and then the sequential presentation of the visual items. Recall was prompted one second after the completion of the visual sequence. During recall, participants attempted to recreate the sequence of six letters that had concluded the list by using a mouse to make selections from the set of nine English letters comprising each list. Participants were instructed to click a given letter no more than once in the recall sequence (since there were never repetitions in the final six items) and to click a *blank* button in place of any forgotten letter, so that correct serial position could be maintained. As each letter was selected, that letter (or a blank) was shown at bottom of the screen. At any time during recall, the participant could also clear and re-enter the items (in serial order).

### Results and Discussion

Crossing rate of presentation and sound conditions, there were four trial types in the RMS task: slow-paced trials with and without irrelevant background sounds and fast-paced trials with and without irrelevant background sounds. Responses were scored as accurate only when a letter was placed in the correct serial position in relation to the end of the list. Accuracy was highest in the quiet slow-paced trials (*M* = 3.321 items, *SE* = 0.210), followed by the IS slow-paced trials (*M* = 2.958 items, *SE* = 0.214), quiet fast-paced trials (*M* = 2.338 items, *SE* = 0.179), and IS fast-paced trials (*M* = 2.100 items, *SE* = 0.175).

A 2 × 2 repeated measures ANOVA considered the impact of sound condition (quiet, IS) and speed of TBR item presentation (fast-paced, slow-paced) on the number of items correctly recalled in serial order. A significant main effect of sound condition (*F*(1,22) = 13.189, *p* = .001, partial η^2^ = .375) demonstrated better performance in the quiet than IS trials. A main effect of presentation speed (*F*(1,22) = 22.619, *p* < .001, partial η^2^ = .507) was also obtained, with better performance in the slow-paced than fast-paced trials. Importantly, no interaction was found between sound condition and speed of presentation, suggesting that the irrelevant background sounds had a similar impact on serial recall regardless of whether participants completed the fast-paced or slow-paced versions of the task (*F*(1,22) = .709, *p* = .409, partial η^2^ = .018).

**Figure 2:**
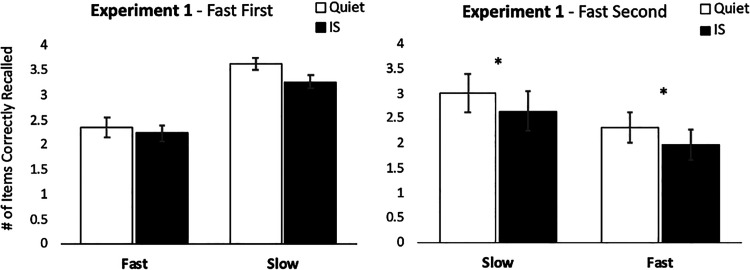
Average number of items recalled in the correct serial position in Experiment 1 for quiet and changing-state irrelevant sound (IS) trials, split for participants who completed the first block of the running memory span task under fast presentation conditions (Fast First, left panel), and those who completed the first block of the task under slow presentation conditions followed by a block with fast presentation (Fast Second, right panel). A qualitatively similar pattern was obtained for each condition order group, but a significant irrelevant sound effect emerged only among the subgroup who started in the slow condition before proceeding to the fast condition. Error bars show standard error of the mean. *p < .05.

If rehearsal was necessary for the ISE, then we would expect to find a substantially diminished ISE in the fast-paced condition, which is least conducive to the use of rehearsal. To the contrary, we observed a significant ISE even in the fast-paced trials, in apparent contradiction of the notion that rehearsal is necessary to produce an ISE. Further inspection of the data did, however, indicate that the order in which participants encountered the different presentation rate blocks may have influenced the size of the effect. Specifically, subjects who first attempted the task with a fast rate of presentation demonstrated a very modest decline in performance during IS trials, whereas those who encountered the faster rate of presentation in the second block showed a more sizable effect (Figure 2). To test the significance of this pattern, we conducted an additional repeated measures ANOVA with sound (quiet, IS) and speed (fast-paced, slow-paced) as within-subjects factors, and block order (fast first, fast second) as a between-subjects factor. This test revealed no main effect of order (*F*(1, 21) = 1.024, *p* = .323, partial η^2^ = .046) and no significant interactions between order and sound or speed. Nevertheless, we conducted a further experiment (Experiment 2) to further rule out the possibility that the group who completed the slow-paced block first may have carried over a rehearsal strategy into the subsequent fast-paced block.

## Experiment 2

Experiment 2 had two aims: first, to replicate the presence of an ISE in a fast-paced variant of the RMS task and, second, to rule out the possibility that the significant ISE obtained in Experiment 1 in association with fast-paced presentation arose from the continuation of a rehearsal strategy induced (in half of the participants) by prior exposure to the slow-paced variant of the task.

Experiment 2 exactly mirrored the procedures used in Experiment 1, except that participants performed only the fast-paced version of the task for two successive blocks and never experienced the slow-paced version. By having participants complete the fast-paced variant twice in succession, this experiment also allowed us to observe the effect of task practice without confounding from experience with the slower condition. To assay participants’ subjective experiences in the task, we also asked participants to complete a brief strategy choice questionnaire.

### Method

#### Participants

Twenty-six native English-speaking, Temple University undergraduates (*M*_*age*_ = 19.65, 17 female) who reported normal or corrected-to-normal vision and hearing, and unimpaired use of their dominant hand participated in exchange for course credit. All subjects gave written informed consent.

#### Procedure

The procedure used in Experiment 2 was identical to Experiment 1 with three exceptions. First, participants completed six practice trials and 40 experimental trials. Second, participants completed two fast-paced (three letters per seconds) blocks of the task. Finally, after completing the task, participants filled out a short strategy questionnaire. The questionnaire asked participants to indicate whether they (1) tried to repeat the letters over and over in order in their minds (i.e., rehearsal), (2) tried to group the letters with other letters (i.e., chunking), (3) simply tried to concentrate or focus, or (4) used a strategy that was not described by one of the preceding alternatives (other).

### Results and Discussion

We first sought to determine whether the size of the ISE differed between the first and second blocks. Once again using accuracy of serial order recall as the dependent outcome, we conducted a repeated measures ANOVA with sound condition (quiet, IS) and block (first, second) as within-subjects factors. This test indicated a significant main effect of sound condition (*F*(1, 25) = 6.953, *p* = .014, partial η^2^ = .218), a marginal effect of block that did not reach significance (*F*(1, 25) = 3.378, *p* = .078, partial η^2^ = .119), and no sound by block interaction (*F*(1, 25) = 2.542, *p* = .123, partial η^2^ = .092).

**Figure 3:**
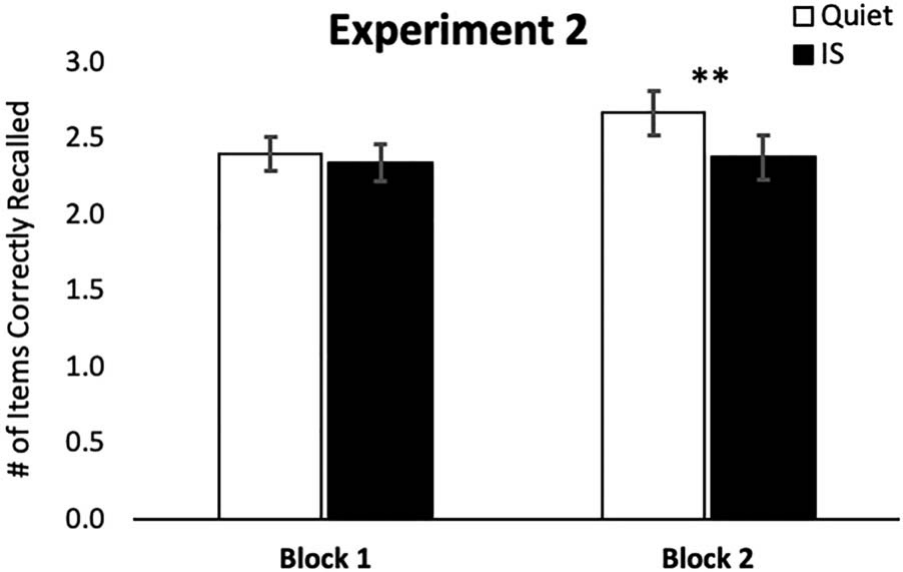
Average number of items recalled in the correct serial position in Experiment 2 in the quiet and changing-state irrelevant sound (IS) trials, split by block. While there was no apparent effect of IS condition on Block 1 performance, a highly significant irrelevant sound effect was obtained for Block 2 due to an apparent increase in performance associated with quiet trials that was not present for the irrelevant IS trials. Error bars show standard error of the mean. *p < .05, **p < .005.

Despite the absence of an interaction in the ANOVA, planned contrasts did indicate a weaker and nonsignificant ISE for the first block (*t*_(25)_ = 0.574, *p* = .571, *d* = 0.73), but a strong and significant ISE for the second (*t*_(25)_ = 3.269, *p* = .003, *d* = 2.78). This overall pattern paralleled that observed for the between-subjects manipulation of block order in Experiment 1 (which also showed a small and nonsignificant ISE when the fast-paced condition constituted the first block, but a larger and significant ISE when the fast-paced condition constituted the second block). A closer look at the data from the present experiment (Figure 3) indicated that this pattern was driven by performance increases across the two blocks for the quiet trials (Block 1 *M* = 2.404, *SE* = 0.109, Block 2 *M* = 2.673, *SE* = 0.145), but essentially flat performance for the two blocks of the IS trials (Block 1 *M* = 2.342 items, *SE* = 0.124, Block 2 *M* = 2.381, *SE* = 0.146). The observation of flat performance across the two blocks for IS trials suggests the possibility that a floor effect might be present in the IS trials. In Experiment 3, we therefore enacted a slightly different recall procedure that has previously been shown to produce better overall performance in the RMS task (Bunting et al., 2006).

After completing the task, participants also filled out a strategy questionnaire asking them to report the method they used to remember the letters during the task. Of note, and somewhat to our surprise, despite the fast-paced presentation rate in this experiment, and the RMS task’s use of long and unpredictable list lengths, nearly half of the participants (*n* = 10) still reported that rehearsal was their primary strategy, while the other half reported primary use of an alternate strategy.

To clarify the potential importance of a rehearsal strategy in producing the ISE, we inspected the data from those individuals reporting that they used rehearsal as their primary strategy relative to those for individuals reporting any one of the other three “nonrehearsal” strategies (chunking, focus, and other). If reliance on rehearsal was an important source of the ISE, then we could expect a relatively larger ISE among participants who reported rehearsal as a primary strategy. Despite the limited sample size remaining in each independent strategy group, a repeated measures ANOVA on the Block 2 performance data, treating sound (quiet, IS) as a within-subjects factor and strategy (rehearsal, nonrehearsal) as a between-subjects factor, again confirmed a highly significant main effect of sound condition (*F*(1, 24) = 10.591, *p* = .003, partial η^2^ = .306), but provided no indication at all of a main effect of strategy (*F*(1, 24) = 0.047, *p* = .831, partial η^2^ = .002) or an interaction between strategy and sound condition (*F*(1, 24) = 0.042, *p* = .393, partial η^2^ = .002). Thus, the size of the ISE did not appear to be dependent on the strategy reported by participants. Because no differences emerged among the two strategy groups, the findings from this experiment offer further evidence against a rehearsal-disruption account. Of course, splitting the already modest sample into two strategy subgroups (with 10 and 16 individuals, respectively) could simply have resulted in an underpowered test of strategy-dependent differences in the ISE. This potential power limitation is an issue we ultimately return to address in Experiment 4.

## Experiment 3

The changing-state effect is hallmarked by the observation that acoustically changing irrelevant stimuli (e.g., k, h, q, and v) produce a particularly strong ISE on task performance, while steady and temporally unchanging irrelevant stimuli (e.g., v, v, v, and v) have a weaker impact on performance (Jones, et al., 1992; Jones & Macken, 1993; Kattner & Ellermeier, 2020). Experiments 1 and 2 of the present study demonstrated that, with an acoustically changing IS stream, the ISE persists even when the task makes serial rehearsal unlikely and that the effect is of comparable magnitude among those who do, and do not, self-report the use of rehearsal.

Experiment 3 was designed to address two specific limitations of the two earlier experiments. First, to better connect the present findings from an RMS to the broader literature on the ISE, Experiment 3 included intermixed trials of an additional “steady-state” IS condition. Thus, RMS performance was tested under three sound conditions: quiet, acoustically changing ISs, and acoustically steady ISs. Second, to try to address possible floor effects that may have partially masked additional impacts on performance in the first two experiments, Experiment 3 adopted a reporting procedure that encouraged participants to recall only the number of end-of-list items that they thought they could recall successfully, rather than specifically the final six items.

### Method

#### Participants

Twenty-four native English-speaking, Temple University undergraduates (*M*_*age*_ = 19.83, 19 female) reporting normal or corrected-to-normal vision and hearing, and unimpaired use of their dominant hand participated in exchange for course credit. All subjects gave written informed consent.

#### Procedure

As in Experiment 2, Experiment 3 once again required participants to complete two blocks of the fast-paced RMS task. However, Experiment 3 included an additional steady-state IS condition for one-third of the trials. The steady-state sound sequences had the same basic structure and parameterization as the changing-state sequences, with the exception that one digit (1, 2, 3, or 4) was repeated auditorily throughout the stream. For each of the two task blocks, participants completed 20 trials under each sound condition (for a total of 60 trials per block), presented in randomized order within the block.

Experiment 3 also utilized a different reporting method than was used in Experiments 1 and 2. Rather than asking participants to specifically recall an item (or indicate a blank) for each of the six final letter positions, participants were instead prompted to recall as few, or as many, of the final six letters as they could (e.g., a participant could initiate recall only at the third-to-last item). This change was instituted to prioritize the correct identification of potentially fewer than six items while limiting output interference that might arise during the attempted retrieval of items (likely to be less well-represented) from positions earlier in the sequence.

### Results and Discussion

A 3 × 2 repeated measures ANOVA crossing sound condition (quiet, steady-state, changing-state) by block (first block, second block) found a significant main effect of sound condition (*F*(2, 40) = 6.206, *p* = .004, partial η^2^ = .237), but no main effect of block (*F*(1, 20) = .290, *p* = .596, partial η^2^ = .014) or interaction between sound and block (*F*(2, 40) = .727, *p* = .489, partial η^2^ = .035). Given the absence of main or interactive effects for block, we collapsed across the two blocks for the remainder of the analyses in this experiment. Planned contrasts of the alternative sound conditions revealed a significant difference in serial recall between quiet (*M* = 2.626, *SE* = 0.117) and changing-state sound (*M* = 2.371, *SE* = 0.121) trials (*t*_(20)_ = 3.381, *p* = .003, *d* = 0.466), and between quiet and steady-state (*M* = 2.462, *SE* = 0.113) trials (*t*_(20)_ = 2.337, *p* = .030, *d* = 0.311), but no significant difference between the steady- and changing-state sound (*t*_(20)_ = 1.219, *p* = .237, *d* = 0.168) trials.

The results of this experiment provide yet another demonstration that irrelevant background sounds produce significant disruption of RMS task performance, even when the rate of presentation should exceed the speed at which rehearsal can be effectively deployed. Moreover, the findings replicate a pattern that has become more prominent in the literature on the ISE. Namely, even nominally steady-state sound streams comprising repeated but discontinuous sound tokens can lead to significant disruption of WM task performance (Bell et al., 2019a; Kattner & Ellermeier, 2020). Moreover, since obligatory engagement of internal seriation processes is not typically assumed for steady-state sequences of the type used in this experiment, the observation of a steady-state ISE offers yet further evidence that this phenomenon is not likely tied to either a rehearsal strategy (which is rendered impracticable by task characteristics) or seriation (which would be potentially signaled by a stronger changing-state than steady-state effect).

Of course, the failure to observe greater disruption in the changing-state relative to steady-state trials does differentiate the present findings with the RMS task from many previous studies showing a reliable difference between these two conditions in other WM tasks. One possibility that should be considered is that the lack of difference between the two sound conditions might again be attributable to floor effects; namely, the changing-state condition would have more strongly impacted performance were it not for the fact that participants reached the floor of performance under these task demands. Despite our intent to lift average performance levels by using revised retrieval instructions in this experiment, overall serial recall accuracy was still poor (fewer than 2.5 items recalled on average even in the quiet condition) and qualitatively similar to that observed in the prior two experiments. Another possibility is that, because the same four digits were repeated in the changing-state IS stream, there simply was not enough *change* to differentiate the changing-state sound effect from the steady-state sound effect; we return to this issue in the general discussion.

## Experiment 4

**Figure 4:**
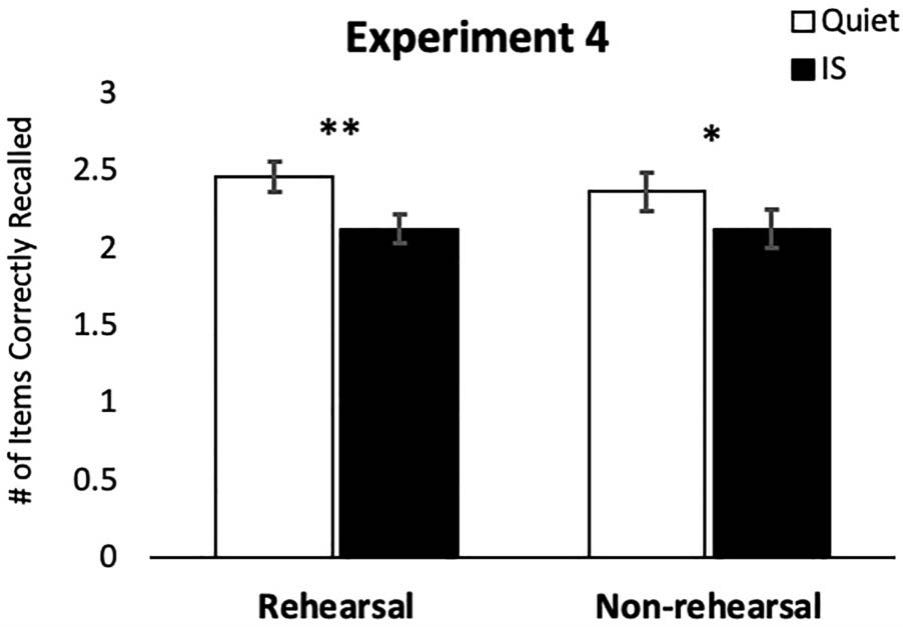
Average number of items recalled in the correct serial position in Experiment 4 for the quiet and changing-state irrelevant sound (IS) trials, split by strategy group. In Experiment 4, a significant irrelevant sound effect emerged both for individuals who reported primary use of a rehearsal strategy and for those who reported primary use of a nonrehearsal strategy. Error bars show standard error of the mean. *p < .05, **p < .005.

**Figure 5:**
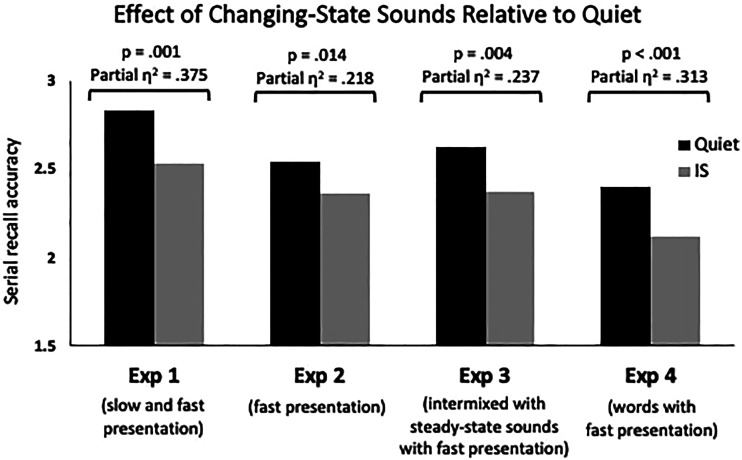
Summary of the effect sizes for the comparison of changing-state irrelevant sound (IS) to the quiet condition in Experiments 1 through 4.

Experiment 4 was primarily intended to explore whether the ISE observed in the prior experiments using simple letter stimuli could be extended to an RMS task using word stimuli, which might elicit a broader range of internal representational codes and might therefore evoke a wider assortment of subject strategies (e.g., strategies based on semantics and mental imagery; Morrison et al., 2016). Specifically, we repeated the same fast-paced RMS task and sound conditions that were used in Experiment 2, but modified the TBR items from letters to common English words (e.g., beach, frame, and mine), and adopted the same reporting procedures used in Experiment 3. Expecting that these stimuli could evoke a more expansive set of encoding and maintenance strategies, we also administered an elaborated (relative to that used in Experiment 2) strategy questionnaire upon completion of the second round of the task. This expanded strategy questionnaire described 11 different strategies that have previously been implicated in WM tasks using word stimuli (Hughes & Marsh, 2020; Morrison et al., 2016; Dunlosky & Kane, 2007; see the *Supplementary Materials* for the questionnaire). Despite the seeming disutility of a rehearsal strategy in the fast-paced RMS task, based on the outcomes of Experiment 2, we anticipated that rehearsal would be likely to emerge as a prominent strategy, but with a larger sample, we hoped that other nonrehearsal strategies would be well-represented among the group. Moreover, to address the potential limitations to statistical power that may have constrained our ability to observe strategy-dependent performance differences in Experiment 2, we also substantially increased the overall sample size for this fourth experiment (Figures 4 and 5).

### Method

#### Participants

Ninety-six Temple University undergraduates (*M*_*age*_ = 19.42, 67 females) with normal or corrected-to-normal vision and hearing participated in exchange for course credit. All subjects gave written informed consent.

#### Stimuli

The words serving as TBR stimuli in Experiment 4 were English words selected from the MRC Psycholinguistic Database (Wilson, 1988), and met the following criteria: one syllable, six letters or less, written frequency of at least 50, and imageability score of at least 500. Fifty-four words were selected from the database, which were then divided into six lists of nine words each. The nine words comprising each list were selected to be both phonologically and semantically distinct from each other. Each trial of the RMS task randomly sampled one of the six lists, and the nine words in that list were then sampled randomly, with replacement, to produce a sequence of 12–20 words (with no repetition occurring in the last six words).

#### Procedure

The procedure used in Experiment 4 was identical to that in Experiment 2, with three exceptions. First, the TBR stimuli constituted a larger pool of commonly encountered English words, instead of a small, fixed, pool of letters. Second, the reporting procedure used in Experiment 3 was adopted for Experiment 4. Third, at the end of the second block, participants completed a memory strategy questionnaire in which they selected the strategy or strategies that most closely described the one(s) they had used most consistently during the task. The questionnaire described a total of 11 different strategies, and participants were instructed to first select a primary strategy, with the opportunity to subsequently select additional (i.e., secondary and tertiary) strategies they may have used. Alternate versions of the questionnaire were used across participants to randomize the order in which each potential strategy appeared in the list.

### Results and Discussion

We first sought to replicate the analyses of Experiment 2 to determine whether the size of the ISE differed between the first and second block when words (rather than letters) functioned as TBR content. Once again using accuracy of serial order recall as the dependent outcome, we conducted a repeated measures ANOVA with sound condition (quiet, IS) and block (first, second) as within-subjects factors. As seen in Figure 4, this test indicated a significant main effect of sound condition (*F*(1,95) = 43.316, *p* < .001, partial η^2^ = .313). Unlike in Experiment 3, but consistent with Experiment 2, there was also a significant main effect of block (*F*(1,95) = 13.366, *p* < .001, partial η^2^ = .123), with performance being better overall in the second block. There was no sound by block interaction (*F*(1,95) = 1.115, *p* = .294, partial η^2^ = .012), suggesting that the ISE was comparable across the first and second blocks.

We next turned our attention to evaluating performance as a function of strategy. Overall, participants remembered more items in their correct serial order during quiet trials (*M* = 2.403, *SE* = 0.076) than for changing-state IS trials (*M* = 2.119, *SE* = 0.070). Fifteen participants were excluded from further strategy-based comparisons due to ambiguous responses on the strategy questionnaire. The breakdown of primary and secondary strategies reported by the remaining 81 participants is shown in Table 1. To evaluate the role of rehearsal in the ISE, we adopted an approach from Hughes and Marsh (2020) and dichotomized participants into rehearsal (n = 46) and nonrehearsal (n = 35) groups. A 2 × 2 repeated measures ANOVA crossing sound condition (quiet, changing-state) by strategy group (rehearsal, nonrehearsal) indicated a significant main effect of sound condition (*F*(1,79) = 35.994, *p* < .001, partial η^2^ = .313), but no main effect of strategy group (*F*(1,79) = .105, *p* = .747, partial η^2^ = .001) and no interaction (*F*(1,79) = .998, *p* = .321, partial η^2^ = .012). Planned *t*-tests comparing performance in the two sound conditions independently for each strategy group revealed a significant ISE for both the rehearsal strategy group (*M* ISE = 0.336, SE = 0.057, *t*(45) = 5.924, *p* < .001, *d* = 0.532) and the group who used an alternate strategy (*M* ISE = 0.240, SE = 0.081, *t*(34) = 2.962, *p* = .006, *d* = 0.334). The results thus reveal a significantly disruptive effect of changing-state ISs on RMS task performance with visually presented words, with the degree of disruption for those who used a rehearsal strategy and those who did not each evincing this pattern (although the effect is numerically larger among the rehearsal group). As with the prior three experiments, these results fail to compellingly support a rehearsal-disruption account of the ISE. A summary graph of the changing-state effect size obtained across the four experiments can be seen in Figure 5.

**Table 1:**
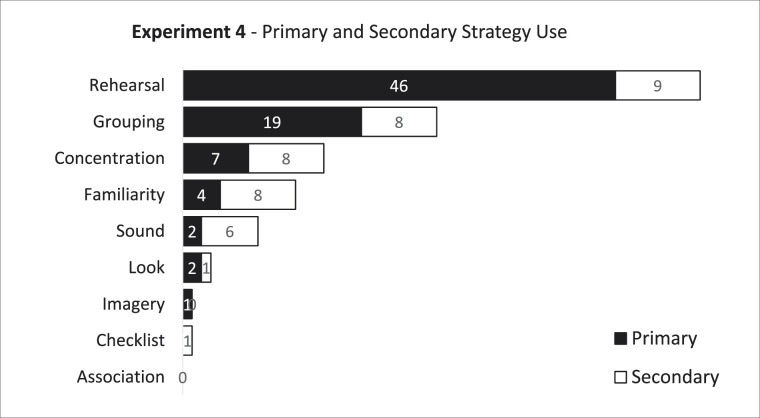
Self-reported primary and secondary strategies used across both blocks in Experiment 4

We next adopted a Bayesian approach to test support for the null hypothesis that the size of the ISE was the same across strategy groups. A Bayesian independent samples *t*-test showed anecdotal evidence in favor of the null hypothesis that those who rehearsed were no more impaired by ISs than those who did not rehearse (*BF*_01_ = 2.785). While we are hesitant to make strong claims based on this level of evidence alone, we feel that along with the frequentist statistics in Experiment 4 and the results from the prior three experiments, the overall evidence fails to support a rehearsal-disruption account of the ISE.

## General Discussion

According to rehearsal-disruption accounts of the ISE, when an irrelevant changing-state sound stream is present in the background, only individuals who persist in engaging a rehearsal strategy during a WM task should evince an ISE. In the present study, we sought to test this class of explanations by determining whether an ISE is present for the RMS task – a WM task that requires ordered output of memoranda, but which should, especially under speeded conditions, discourage the use of a serial rehearsal strategy.

Across four experiments, we show that the ISE is a reliable phenomenon in the RMS task when verbal materials serve as the TBR stimuli. In Experiment 1, we demonstrated that an ISE occurs in both slow-paced (presumably more conducive to rehearsal) and fast-paced (presumably less conducive to rehearsal) versions of the task. In Experiment 2, we showed that an ISE persists even when there could not be inducement to rehearse due to prior exposure to a slow-paced version of the task. In Experiment 3, we found that the phenomenon is present for both changing-state and steady-state sound streams. In Experiment 4, we found that the effect of changing-state ISs persists when words serve as the TBR stimuli and that the size of the ISE is comparable for participants who report using rehearsal as their primary strategy and those who report using an array of alternate, nonrehearsal, strategies.

According to the changing-state hypothesis and other interference-by-process accounts (Beaman & Jones, 1997; Hughes & Jones, 2005; Jones, 1994; Jones et al., 1992, 2010; Jones & Tremblay, 2000; Macken et al., 1999; Marsh et al., 2009), changing-state sound sequences are processed preattentively for order, which interferes with the similar ordered maintenance process that occurs during rehearsal. Since steady-state sound streams should not be processed for order, they should not conflict with the ordered maintenance or retrieval of TBR items. Consequently, changing-state sequences should be significantly more disruptive to performance than steady-state streams. The findings from the current study do not support either of these assertions. In particular, in Experiments 2 and 4, we found a comparable deficit in performance across individuals who engaged in rehearsal and those who did not, and in Experiment 3, we found equivalently disruptive effects of changing-state and steady-state sounds. Taken together, these findings are not in alignment with rehearsal-disruption accounts.

While the present study was not designed to proffer direct support for alternative explanations for the ISE, accounts that emphasize general attentional mechanisms do provide a relatively straightforward way to interpret the overall pattern of findings. Broadly, such accounts describe the ISE as a result of attentional shifts away from the primary memory task and toward the IS stream, which decreases the attentional resources available for maintenance of TBR items. In the RMS task, as with most other WM tasks, the transient withdrawal of attention away from internal representations of the TBR sequence could lead to a weaker trace representation for the items and their sequencing, and thus poorer performance. In one fairly recently specified attention-disruption model, the graded attentional model (Bell et al., 2019a), the magnitude of attentional capture induced by the IS stream is thought to be determined by the degree to which consecutive sound objects match the most recently heard object (because the comparison process of each new sound object to the attentional filter requires some degree of attentional resources but decreases when newly encountered sound objects match the attentional filter object). Accordingly, this model predicts that steady-state sound streams can produce a base level of disruption, while changing-state sounds should be especially deleterious for performance. The model provides a facile explanation for two key discoveries from the present study: First, rehearsal has no special role in producing the ISE, and second, steady-state sounds are sufficient to produce an ISE. However, the apparent equivalence of the steady-state and changing-state effects observed in Experiment 3 is less amenable to this particular account. As we noted earlier, this apparent lack of difference could be explained by power limitations, floor effects, or nuances in the particular construction of the IS streams.

The present evidence can also be accommodated by theories that make no particular reference to either serial rehearsal or attention. The Token-Gradient model (Campbell, 2000; Campbell et al., 2003), for example, makes predictions similar to those of the graded attentional model, without ascribing any particular importance to either rehearsal or attentional processes. Under the Token-Gradient model, each nonrepeating token comprising the IS stream creates an increasingly activated representation in WM, thus forming an activation gradient. The sound token gradient consequently interferes with a similarly established activation gradient representing the successively presented TBR items in a serial recall task, and it is this conflict that leads to the ISE. Bell et al. (2013) offer a further alternative view that ascribes no particular importance to either serial rehearsal or attention, suggesting instead that the ISE might arise due to interference with the processes that bind an item to its context. In their account, items can be bound to any contextually relevant feature, which can include, but is not restricted to, serial order position. This explanation has been applied to the ISE obtained in serial order recall tasks, where a loss of order information is thought to be caused by blockade of the binding of the items to their serial position. The ISE observed in all four of the experiments we conducted, and the absence of a true changing-state effect in Experiment 3, may be made commensurate with this explanation by assuming that the binding of items to their serial position is equally disturbed by any type of IS, even steady-state sounds; however, this assumption is not specifically addressed within Bell et al.’s (2013) work.

While the present study extends prior work exploring the mechanisms that underlie disruption of WM by IS, we acknowledge some limitations that temper the conclusiveness of our results. First, we acknowledge that retrospective self-reports of strategy choice may be a particularly unreliable gauge of actual strategy use, since strategies are difficult to describe (e.g., may have equivocal interpretations); may not be fully accessible to conscious awareness; and may change over the course of task performance. These limitations may explain why, in Experiments 2 and 4, participants report using a serial rehearsal strategy that we had explicitly tried to curtail in the RMS task. Yet, in the two experiments where we assessed strategies, it seems that rehearsal was still prominent in the self-reports, even under the demands of a speeded RMS task. Since we assessed strategies only after completed blocks, it is also possible that participants implemented multiple strategies across trials that were poorly summed up in block-wise strategy assessments. Perhaps even *nonrehearsers* attempted to rehearse during some trials, which thereby created the opportunity for rehearsal-disruption processes to influence the overall performance findings. To address the possibility that occasional rehearsal might have elicited an ISE, we conducted analyses that paralleled those reported under Experiment 4 while sorting out participants who reported using rehearsal as a primary, secondary, or tertiary strategy. This more restrictive approach did not qualitatively change the results (i.e., a significant ISE was still obtained for those individuals who never reported rehearsal). On the contrary, another concern could be that it is not possible for participants to engage in a proper rehearsal strategy in the fast-paced RMS task; thus, those who reported rehearsing were improperly labeling their strategy use. If this is true, and a serial rehearsal strategy was not properly engaged, the fact that we still observed a significant ISE among all participants supports the same conclusion we are presenting here – that serial rehearsal does not have to be engaged to produce an ISE in an ordered recall task in the presence of changing-state sounds.

While retrospective self-report has obvious limitations, the approach we adopted to index strategy use closely parallels other recent work by Hughes and Marsh (2020), which used the same strategy questionnaire and similarly dichotomized participants into rehearser and nonrehearser groups. In that study, performance was tested on a missing-item task, and a changing-state effect emerged among rehearsers but not among nonrehearsers. That pattern of findings, which seems to signal the importance of rehearsal in producing susceptibility to IS conditions, appears to be inconsistent with the present findings. One possible explanation for this apparent inconsistency is that the retrospective self-report strategy assessment method does not adequately capture the specific trial-by-trial processes that participants rely on. Another obvious possibility is that the unique task demands imposed by the RMS and missing item tasks lead participants to interpret the strategy options differently. While the missing-item task might encourage ordered maintenance, it does not require ordered recall. The RMS, meanwhile, has the opposing characteristics of discouraging ordered maintenance strategies but requiring ordered recall. Different interpretation of what *rehearsal* references in these two cases might explain the disparate outcomes. That is, the rehearsal strategy used to produce a nonserial one-item response demanded by the missing item task may be different from the rehearsal used to produce a serial multi-item list (such as that required for the RMS).

A second issue is that memory performance was low overall; across experiments, participants correctly reported an average of fewer than three items (out of a possible six) in the fast-paced RMS task. While we tried to address low performance by adjusting the reporting instructions in Experiments 3 and 4, this change did not produce a change in mean performance levels. Thus, there remains the possibility that floor effects may have confounded certain contrasts. Nevertheless, while the study may have been insensitive to more subtle ISE phenomenon (e.g., the differences between changing and steady state IS), each of the four experiments was still sufficiently powered and above the floor of performance to produce a significant ISE.

The current results demonstrate the disruptive effect that distracting sounds can have on short-term cognitive functioning, as measured in the RMS task. Continued work exploring alternate ways to gauge and manipulate participant strategy use in WM tasks could produce fruitful discoveries. Future work could, for instance, explore the ISE after explicit instructions to engage in a certain strategy, or after allowing participants the freedom to first explore strategies and to then choose one to stick with for the remainder of the experimental blocks. Such future work might allow us to reach even more conclusive determinations regarding the exact role of rehearsal in the ISE and shed further light on the specific conditions that affect the emergence of this phenomenon.

## Notes

**Publication Ethics** All subjects participating in the Experiments 1–4 gave informed, written consent.

**Open Data** Data for all experiments can be accessed at the following link: https://osf.io/7knxa/?view_only=e69036ad012440efbee678358130ee18 (Samper et al., 2021). My materials are not available in full. My article contains no experiment with a completely executed preregistration.
